# Influence of social characteristics on use of paediatric emergency care in Sweden - a questionnaire based study

**DOI:** 10.1186/s12873-018-0210-5

**Published:** 2018-12-27

**Authors:** Julia Ellbrant, Jonas Åkeson, Jenny Eckner, Pia Karlsland Åkeson

**Affiliations:** 1 Department of Clinical Sciences Malmö, Paediatrics, Lund University, Skåne University Hospital, SE-20502 Malmö, Sweden; 2 Department of Clinical Sciences Malmö, Anaesthesiology and Intensive Care Medicine, Lund University, Skåne University Hospital, SE-20502 Malmö, Sweden; 3 Department of Clinical Sciences Malmö, Surgery, Lund University, Skåne University Hospital, Jan Waldenströms gata 11 A, SE-20502 Malmö, Sweden

**Keywords:** Children, Emergency department, Socio-economic status, Triage, Urgency

## Abstract

**Background:**

Parental social characteristics influence the use of emergency departments (ED) in the USA, but less is known about paediatric ED care-seeking in countries with national health insurance. This prospective study was designed to evaluate associations between parental care-seeking and social characteristics, with emphasis on impact of non-native origin, at a paediatric ED in Sweden, a European country providing paediatric healthcare free of charge.

**Methods:**

Parents attending a paediatric ED at a large urban university hospital filled out a questionnaire on social characteristics and reasons for care-seeking. Information on patient characteristics and initial management was obtained from ED registers and patient records. Paediatric ED physicians assessed the medical appropriateness of each patient visit triaged for ED care.

**Results:**

In total, 962 patient visits were included. Telephone healthline service before the paediatric ED visit was less often used by non-native parents (63/345 vs. 249/544, *p* < 0.001). Low-aquity visits, triaged away from the ED, were more common among non-native parents (80/368 vs. 67/555, OR = 1.66; *p* = 0.018), and among those reporting lower abilities in the Swedish language (23/82 vs. 120/837, OR = 2.66; *p* = 0.003). Children of non-native parents were more often assessed by physicians not to require ED care (122/335 vs. 261/512, OR = 0.70; *p* = 0.028).

**Conclusions:**

This study confirms more direct and less urgent use of paediatric ED care by parents of non-native origin or with limited abilities in the Swedish language, proposing that parental social characteristics influence paediatric ED care-seeking, also in a country with healthcare free of charge, and that specific needs of these groups should be better met by prehospital medical services.

**Electronic supplementary material:**

The online version of this article (10.1186/s12873-018-0210-5) contains supplementary material, which is available to authorized users.

## Background

The number of emergency department (ED) visits is growing rapidly all over the world [1, 2]. Although parental concern is most important for a child to attend a paediatric ED in due time for an urgent medical condition, medical severity is perceived differently by parents [3]. Assessing and supervising a sick child while also navigating a complex medical system may be challenging to parents, and many of them seek paediatric ED care also for less urgent medical conditions [4–6]. Evaluating care-seeking patterns of parents might facilitate more optimal management of their children at adequate medical levels of healthcare, while also promoting rapid and appropriate management of those children requiring urgent paediatric ED care [6].

Higher use of adult ED care by immigrants has been reported in different parts of Europe [7–10]. We recently found direct seeking of paediatric ED care (with no previous medical consultation) to be more common from city districts with higher proportion of immigrants and lower socio-economic status [6]. In the USA, those circumstances have been reported to be strongly associated with overall poor general access to healthcare [11–17], and also been considered to result mainly from lack of national insurance. Foreign born citizens more often have lower socioeconomic status than native borns in European countries like Sweden, considering both employment [18, 19], income [20], and education [21], However, little is known about how social characteristics of parents influence their use of paediatric ED care in healthcare systems based on national public insurance. Such information would be most useful to improve access for sick children to adequate levels of emergency care, and also to identify and address barriers to prehospital medical services.

Based on our previous findings of different paediatric ED seeking patterns between parents from city districts with higher or lower proportions of non-native inhabitants [6], we designed this prospective study primarily to evaluate associations between parental origin and abilities in the the Swedish language, corrected for other social characteristics, and paediatric ED care-seeking, triage and management at a large urban university hospital in a European country providing paediatric healthcare free of charge.

## Methods

### Study design

This cross-sectional, prospective, questionnaire study, approved by the regional Human Research Ethical Review Board, Lund, Sweden, was carried out at Skåne University Hospital in the city of Malmö in southern Sweden during a four-week study period in February and March, to reflect patient high-load conditions, corresponding to approximately two-thousand paediatric ED visits per month.

### Study setting

At the time of the study, the university hospital provided primary healthcare within a catchment area of approximately 400,000 inhabitants, including 19% children [22]. The city population, varying considerably in socio-economic status (education, employment, income, social assistance), comprised 31% first-generation (both parents born abroad), and another 11% second-generation, immigrants from 177 countries [22].

In Sweden, paediatric healthcare, including ED care, is provided free of charge. Children are enlisted at child healthcare centres, responsible for regular health check-ups and parental education until the age of five, as well as at healthcare centres providing access to general physicians for both scheduled and more urgent medical care. There are few private options within paediatrics or general practice. Parents are expected to seek medical advice at healthcare centres during daytime or through the national telephone healthline system, available twenty-four seven, for their sick children except for in urgent cases. During evening-time and weekends (also including day-time), a primary care walk-in-centre is available approximately 1 km from the hospital. The paediatric ED provides care of infants and children for all kinds of medical emergencies with no demand for professional referral. Paediatric ED patients with surgical, orthopaedic, or serious otorhinolaryngeal, conditions are primarily referred directly by nurses to the adult ED next-door for further evaluation.

### Study patients

Up to 17-year-old patients arriving at the paediatric ED between 08:00 and 21:00 during the study period were considered eligible for inclusion. For practical reasons, nighttime patients (21:01–07:59) could not be included. Scheduled revisits for rapid check-up of recent ED visits were excluded, as were children arriving alone or (a few) critically ill.

Written information about the study in Swedish, English or Arabic, and corresponding oral information in Swedish or English, was provided to parents and to children above 7 years of age. Signed informed consents were obtained from all participating parents, and from all study patients above 15 years of age.

On ED arrival, an experienced paediatric nurse assessed each study patient to determine the appropriate level of medical care, based on structured routine assessments of patient history, presenting symptoms, and clinical signs, according to the established and locally implemented Rapid Emergency Triage and Treatment system (RETTs) [23, 24], and the Emergency Signs and Symptoms (ESS) system [25]. Each study patient was accordingly triaged to be assessed by an ED paediatrician, referred to another healthcare provider, or triaged to return home with medical advice. All patients triaged to an ED paediatrician were also scored for medical urgency, according to a four-level Likert scale, to be managed immediately (level 1), not immediately but within an hour (level 2), within one to 3 h (level 3), or within more than 3 h (level 4).

Since native citizens in the Nordic countries speak similar languages and have resembling healthcare and hospital systems, it seems reasonable to assume that parents from those countries have similar abilities in navigating the Swedish healthcare system. Thus, parents of the study patients were primarily evaluated with respect to national origin, by comparing those born in a Nordic country, referred to as native parents, with those born in other parts of the world, referred to as non-native, and with respect to abilities in the Swedish language.

### Questionnaire and protocol

All parents of eligible study patients, were asked to fill out a questionnaire (Additional file 1) on their recent medical contacts, reasons for seeking ED care, country of birth, self-rated abilities in the Swedish language, educational level, current employment, civilian status, number of children, and their sick child’s order among siblings. Interpreters required for medical evaluation also assisted in filling out questionnaires. Statistical analyses for each separate variable were based on the parent with the individually highest rated levels of education, employment, and abilities in Swedish.

Study patients assessed by ED physicians were also evaluated, according to a structured protocol (Additional file 2), to state whether ED care, primary care or no healthcare would have been the individually most appropriate level of medical care, and also how medically urgent the ED visit was considered to have been. The physicians at the paediatric ED included senior specialists, residents and interns, as recorded in the physicians’ protocol, however with more experienced doctors available as backup for those with less experience.

The questionnaire and the protocol were both carefully designed according to the specific study settings and purposes. They were then tested and evaluated during a two-day pre-study period at the ED to detect and minimize potential risks of misunderstanding.

### Statistics

The Statistical Package for the Social Sciences (SPSS) for Windows, version 24.0 (IBM Corp., Armonk, California, USA), was used to record, structure and analyze study data.

Proportions were compared with the two-sided Fisher’s exact test. Univariate and multiple binary logistic regression analyses were used to evaluate associations between categorical data, and results are reported as odds ratio (OR) with 95% confidence interval (CI). Probability (*P*) levels at < 0.05 were considered to indicate statistical significance.

Two predefined multiple regression models – for low-acuity ED visits (triaged away from the ED by nurses), and for high-aquity ED visits (assessed appropriate for ED care by physicians) – were designed, based on six predefined variables of parental social status, and also on patient gender and age, without screening for statistical significance.

## Results

**Fig. 1:**
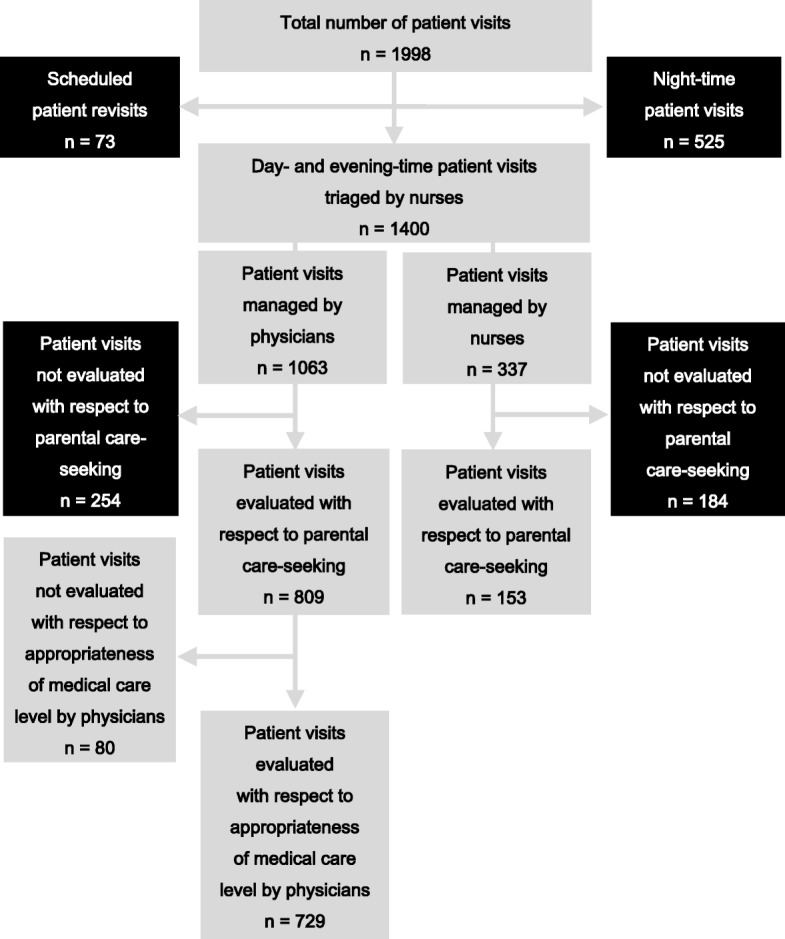
Initial triage and assessed medical appropriateness of patient visits at a paediatric emergency department (ED) of a large urban university hospital in southern Sweden

During the four-week study period, 598 (525 nighttime, 73 scheduled) of 1998 paediatric ED visits (30%) were considered non-eligible for inclusion (Fig. 1). Out of 1400 eligible study patients we included 962 patients – 809 of 1063 eligible day- or evening-time patient visits (76%) triaged to, and 153 of 337 eligible patient visits (45%) triaged away from, the ED.

Included and non-included patients triaged to or from the ED on arrival did not differ significantly in age or gender, and neither did children of parents responding or not responding to parts of the questionnaire. The range of internal loss of answers to single questions was 3.3–8.9% (Additional file 3: Table S1).

The protocol was completed by ED physicians (29% specialists, 42% residents, 29% interns) in 729 study patients (90%) triaged for ED care, and not differing in age, gender or initial medical priority from those 80 study patients (10%) with no such assessments.

Both parents of 368 included children were born outside a Nordic country – 43% in the Middle East, 34% in other parts of Europe, and 14% in Africa. Native parents of 30 out of 555 study patients were born in Denmark, and the others in Sweden.

Characteristics of study patients and their parents, according to appropriateness of ED care as assessed by nurses and physicians, are reported in Table 1. Forty-five percent of the patients triaged for ED care were assessed more suitable for primary care. All patients initially triaged to require immediate medical attention, and later assessed as more suitable for primary care, were above 6 months of age and had no more than one deviating vital parameter. In contrast, corresponding patients later assessed as more suitable for ED care had two or more deviating vital parameters, were more often assessed to require medical attention within 1 h (10/17 vs. 2/11, *P* = 0.054), and were also more frequently admitted for hospital care (10/17 vs. 0/11, *P* = 0.002).

**Table 1:** Characteristics, initial triage and medical appropriateness of patient visits at a paediatric emergency department (ED) of a large urban university hospital in southern Sweden

	Paediatric emergency department (ED) visits initially triaged				
	home or for other level of care (*n* = 153)		to ED physician and later assessed appropriate for primary care(*n* = 331)	to ED physician and later assessed appropriate for ED care (*n* = 398)	
	Number (%)	*P*-value^a^	Number (%)	Number (%)	*P*-value^a^
Age (years)					
0–0.5	18 (12)	< 0.001	62 (19)	126 (32)	< 0.001
0.6–1	56 (37)	> 0.300	118 (36)	124 (31)	0.153
2–6	62 (41)	0.007	111 (34)	100 (25)	< 0.001
7–12	12 (8)	> 0.300	25 (8)	31 (8)	> 0.300
13–17	5 (3)	> 0.300	15 (5)	17 (4)	> 0.300
Female gender	67 (44)	0.287	174 (53)	180 (45)	0.198
Main presenting problem					
Vomiting/Diarrhoea	39 (25)	0.081	50 (15)	72 (18)	> 0.300
Fever	32 (21)	> 0.300	68 (21)	81 (20)	> 0.300
Breathing problem	6 (4)	< 0.001	44 (13)	68 (17)	0.004
Cough/Runnning nose	22 (14)	0.207	46 (14)	32 (8)	0.005
Neurological problem	0	0.146	3 (1)	11 (3)	0.014
Other	54 (35)	> 0.300	120 (36)	134 (37)	> 0.300
Triaged to be assessed					
immediately	–	–	11 (3)	17 (4)	> 0.300
within 1 h	–	–	110 (33)	149 (37)	0.245
within 1–3 h	–	–	137 (41)	200 (50)	0.017
within > 3 h	–	–	73 (22)	32 (8)	< 0.001
Hospital admission	–	–	1 (< 1)	75 (19)	< 0.001
Direct seeking^b^	73 (52)	0.005	128 (42)	132 (36)	0.008
Both parents born outside Nordic countries^b^	80 (54)	< 0.001	133 (42)	122 (32)	< 0.001
Both parents having low^c^ understanding of Swedish^b^	23 (16)	0.004	34 (12)	21 (5)	< 0.001
Both parents educated less than 12 years^b^	59 (41)	> 0.300	139 (44)	140 (36)	0.057
Both parents currently unemployed^b^	28 (19)	0.070	52 (16)	43 (11)	0.014
Single-parent household^b^	14 (10)	0.144	57 (18)	47 (12)	> 0.300
First-born child^b^	89 (65)	0.008	166 (55)	196 (52)	0.067

^a^Obtained by statistical comparison with the other categories of study patients

^b^According to parental questionnaire with 3.3–8.9% lack of responses to single questions

^c^Self-rated as levels 1–4 on a six-level Likert scale

Study patients triaged away from the ED more often arrived directly, i. e. with no previous medical consultation, than those triaged for assessment by an ED paediatrician (73/140 vs. 286/736, *P* = 0.004). Infants below 6 months were more often assessed suitable for ED care (126/206 vs. 272/676, *P* < 0.001) than the older children, whereas children aged 2–6 years were more often triaged away from the ED (62/300 vs. 91/571, *P* = 0.008), compared with other age groups.

After adjustments for patient age and gender, country of birth, educational level, employment, self-rated abilities in the Swedish language, civilian status, number of children, and child’s order among siblings, there were strong statistical correlations between both parents being non-native, and direct seeking of ED care (179/332 vs. 168/347, OR = 2.23; *P* < 0.001), as well as less use of the telephone healthline service before the ED visit (249/544 vs. 63/345, P < 0.001) compared to native parents.

Being triaged away from the ED was statistically associated with having non-native parents (80/368 vs 67/555, OR = 1.66; *P* = 0.018) or parents with low understanding of Swedish (23/82 vs. 120/837, OR = 2.66, *P* = 0.003), and also with being first-born (89/479 vs. 47/411, OR = 1.96; *P* = 0.001) (Table 2). Corresponding number of patient visits and missing values to multiple regression analyzes in Table 2 are reported in Additional file 3: Table S1.

**Table 2:** Adjusted multiple logistic regression models on paediatric emergency department (ED) visits triaged away from the ED or assessed appropriate for ED care at a paediatric ED in Sweden

Parental socal characteristics^a^	Paediatric ED visits initially triaged home or for other level of healthcare		Paediatric ED visits initially triaged for ED care and later also assessed appropriate, for ED care^b^	
	OR^c^ (95% CI^d^)	*P*-value	OR^c^ (95% CI^d^)	*P*-value
Both parents born outside the Nordic countries	1.661 (1.091–2.528)	0.018	0.698 (0.506–0.962)	0.028
Both parents having low^e^ understanding of Swedish language	2.655 (1.399–5.035)	0.003	0.429 (0.228–0.808)	< 0.001
Both parents educated less than 12 years	0.985 (0.650–1.494)	> 0.300	0.863 (0.632–1.177)	> 0.300
Both parents unemployed	0.997 (0.557–1.787)	> 0.300	0.888 (0.553–1.425)	> 0.300
Single-parent household	0.579 (0.302–1.109)	0.099	0.869 (0.554–1.363)	> 0.300
First-born child	1.960 (1.299–2.956)	0.001	0.681 (0.507–0.914)	0.010

^a^According to parental questionnaire with 3.3–8.9% lack of responses to single questions

^b^According to structured assessments by ED physicians

^c^Odds ratio (OR) adjusted for patient age, gender, and order among siblings, for country of birth of the parents, knowledge of Swedish language, level of education, and employment (highest level among parents), and household status, by multiple binary regression analysis

^d^Confidence interval (CI) of OR

^e^Self-rated as levels 1–4 on a six-level Likert scale

Accordingly, patients triaged for ED care with two non-native parents (122/135 vs. 261/512, OR = 0.70; *P* = 0.028), or with parents having low understanding of Swedish (21/78 vs. 365/770, OR = 0.43; *P* = 0.009), were less often considered appropriate for ED care, as were first-born patients (196/451 vs. 184/368, OR = 0.681; *P* = 0.010) (Table 2).

Unemployed parents were less likely to seek with children considered appropriate for ED care (43/123 vs. 343/731, OR = 0.60; *P* = 0.013), and unemployed parents (58/118 vs 291/735, OR = 1.51; *P* = 0.042), and those with less than 12 years of education (153/332 vs. 193/514, OR = 1.37; *P* = 0.030), more often arrived directly. However, those associations were no longer statistically significant after adjustments for ethnic origin and knowledge of Swedish.

Regardless of parental origin and actual level of urgency, there were no correlations between assessments by parents and ED physicians of medical urgency based upon the estimated time limit until physician’s assessment was considered to be required.

## Discussion

This Swedish study at a paediatric ED shows statistical associations between direct and less urgent use of ED care, and parental characteristics, such as non-native origin as well as low abilities in the Swedish language.

Accordingly, non-native inhabitants in different European countries have been found to use ED care more than natives, and also for medically less urgent reasons [26]. An Italian study has reported higher total paediatric ED use and also, in agreement with our findings, more non-urgent visits, by children of non-native parents [8]. We recently found higher use of paediatric ED care from the city district with the highest proportion of non-native inhabitants [6]. Other Scandinavian studies [7, 9] have also reported higher ED use by adult non-natives. In contrast, some American investigators have reported fewer ED and primary care visits by adult immigrants and their children, mainly due to lack of national public insurance and low access to healthcare [11–17]. Accordingly, the American College of Physicians [27] has addressed need for a major change in American healthcare policy to provide adequate medical healthcare to those vulnerable groups.

In the present study, we confirmed an association between direct seeking of paediatric emergency care and medically less urgent ED visits among non-native parents, but we have no information on corresponding primary care visits. Our findings might reflect barriers to prehospital healthcare services for those groups, and problems for the healthcare system to reach them.

That non-native origin of the parents was significantly associated with less prehospital medical contact, after adjustment for low understanding of Swedish, and that their children were more often triaged away from the ED could suggest that cultural differences – not only reflecting linguistic barriers – might influence seeking of paediatric ED care also in a healthcare system based on national public insurance.

Actual organization of medical healthcare services in different countries might, at least in part, explain some differences in healthcare seeking between parents of sick children [28], e. g. whether they primarily turn to public or private, or to primary or hospital-based, healthcare facilities for respiratory illness, diarrhoea or fever [28].

However, that reduced paediatric ED use by non-native parents might be expected over time has been suggested in a recent study, where children of Norwegian first-generation immigrant parents used primary healthcare less, and of second-generation immigrant parents more, than did children of native parents [29]. Accordingly, Latino-families with lower acculturation have been found to use paediatric ED care as a more regular source of healthcare than those having lived longer in the USA [30].

In agreement with our findings, linguistic barriers to communication among Danish [31] and American [32, 33] immigrants have been found to be associated with less use of prehospital services for medical advice or assistance, particularly by telephone. Accordingly, the use of telephone healthline by non-native inhabitants in Sweden has been claimed to complicate medical decision-making [34], and multilingual telephone operator services have been proposed to meet this challenge [32]. Communication problems and lack of confidence have also been reported as major healthcare obstacles by non-native parents in Sweden [35].

Our findings that shorter education and unemployment among parents were associated with more direct ED seeking, and also with more triage away from the ED on arrival – although non-significantly when adjusted for ethnic origin and knowledge of Swedish – conform to more non-urgent use of paediatric ED care by American parents with lower levels of educational and economical status [36].

Non-native and low social status populations have been found to have lower health literacy [37, 38], i.e. individual ability to obtain, process and understand basic health information and services required for appropriate health decisions [39]. Low health literacy has also been identified as a predictor of more frequent and less urgent paediatric ED use in the USA [40, 41]. Accordingly, adult non-native ED patients in Norway were recently reported to rate their medical level of urgency higher than native ones, regardless of physicians’ assessments [42]. Some American interventions to improve health literacy and care-seeking behaviour have been claimed to reduce paediatric ED visits of non-native [43] and low social status [44] parents, indicating that inadequate knowledge of the local healthcare system might be preventable by early interventions bridging over barriers to navigating the system. Low health literacy and low socio-economic status might both reflect risks of delayed healthcare and poor health status [12–14] – challenges even more important to manage.

Our finding that parents attending with their first-born child were more often triaged away from the ED, and also less often considered appropriate for the ED by physicians, could reflect their lower experience as parents and thus higher need for medical advice and re-assurance. Accordingly, first-time parents have been reported to be more worried [45], and to use more out-of-office-hour primary care in Denmark [41] and paediatric ED care in France [46]. Additional information at childhealth centres might improve knowledge, and reduce non-appropriate use of ED care, among less experienced parents.

Even though medical conditions of sick children are often considered more serious and urgent by parents than by professionals, as found in the present and previous [47] studies, the concern of parents for their children remains most important for early detection and management of medical conditions*.* Primary care services located close to a paediatric ED [48], and various fast-track solutions [49], might facilitate rapid and appropriate management of sick children whose parents seek paediatric ED care regardless of other available alternatives.

That approximately one fourth of patients in the present study were triaged on arrival not to require paediatric ED care, confirming previous findings [5], highlights the importance of rapid and medically safe initial assessment of each child, according to defined [23, 24] criteria, as well as medical advice and re-assurance by experienced nurses [5], particularly when there is no demand for professional referral to the ED. In addition, the fact that several study patients initially triaged for paediatric ED care were later assessed more suitable for primary care might be considered to reflect wide medical safety margins of our triage systems.

A major strength of this study is that all paediatric ED patients within a catchment area of approximately fourhundred thousand attended the same single hospital. Although the study was carried out at a large university hospital, main results on care-seeking could most probably be considered to reflect major Swedish city regions, since population structures and healthcare systems do not differ much between larger urban regions within the country.

The study might have been limited by potential language barriers – also preventing some parents from participating – although interpreters required for individual ED management assisted in filling out the questionnaires. However, our main results would rather have underestimated the impact of language barriers if parents of patients triaged away from the ED chose not to participate because of low knowledge of Swedish. The parents level of understanding of the Swedish language was also self-estimated which might be a limitation to our results.

Another study limitation is that many parents of children triaged away from the ED, heading for primary care or back home, wished to leave the ED soon after completion of the triage without having to spend more time there, despite strong efforts to include them in the study. However, the lack of differences in age and gender between study participants and non-participants might indicate that study information obtained does reflect patients triaged away from the ED in this respect. Another limitation (despite lack of differences in age and gender between day- and nighttime ED visits) is that we did not have the capacity to enroll late evening- and nighttime ED patient visits.

Finally, since no validated evaluation tools suitable enough for our study purposes were available, we had to design, test, modify and apply study-specific questionnaire tools.

## Conclusions

This Swedish study confirms more direct and less urgent use of paediatric ED care by parents of non-native origin or with limited abilities in a new language, corrected for other variables of social status, despite paediatric healthcare being provided free of charge. Specific needs of these groups should be better met by prehospital medical services to facilitate safe and more rapid management of their children at medically adequate levels of healthcare, and also to promote appropriate management of paediatric patients requiring more urgent ED care.

## Additional files

Additional file 1: Questionnaire on social status and seeking of paediatric emergency care (to be filled out by parents of study patients). (DOC 61 kb)

Additional file 2: Protocol on medical appropriateness of individual patient visits triaged for assessment - (to be filled out by paediatric emergency physicians). (DOC 35 kb)

Additional file 3: **Table S1.** Patient visits and parental social characteristics at a large urban peadiatric emergency department (ED) in southern Sweden. Proportions and corresponding multiple regression analyses are reported in Table 2. (DOCX 24 kb)

## Abbreviations

CI: Confidence interval
ED: Emergency department
HL: Health literacy
OR: Odds ratio
