# Supplementary material for: Influence of social characteristics on use of paediatric emergency care in Sweden - a questionnaire based study
Source: BMC Emerg Med. 2018 Dec 27;18:59. doi: 10.1186/s12873-018-0210-5 (PMC6307227; doi:10.1186/s12873-018-0210-5)
Supplement: Supplementary file 1 — Questionnaire on social status and seeking of paediatric emergency care (to be filled out by parents of study patients). (DOC 61 kb) [file 12873_2018_210_MOESM1_ESM.doc]

**Questionnaire, parents**

**Date: _____________________ Time of arrival: _______________________**

1. Gender of your child?  Girl  Boy
2. Social security code of your child? _________________________
3. Was your child referred to the emergency department?  Yes **** No
4. Did your child arrive by ambulance?  Yes  No
5. I consider my child to be acutely ill (tick the most appropriate box)

**I do not agree at all I agree completely**

     

1. I am very worried about the current health status of my child (tick the most appropriate box)

**I do not agree at all I agree completely**

     

1. I believe that my child needs to be assessed by a physician

 immediately

 within 1 hour

 within 1-3 hours

 within 4-6 hours

 within 6-12 hours

 within 12-24 hours

 within 1-7 days

1. Where do you usually seek healthcare for your sick child?

 I have never sought healthcare for my child

 At the paediatric emergency department

 At a primary care unit

 At a private physician

 Other: ______________________________________

1. Have you been in contact with any healthcare professionals within the last 24 hours before your emergency department visit today?

 No (move on to question 11)

 Yes, most recently with the telephone healthcare line

 Yes, most recently with a primary care unit

 Yes, most recently with a paediatric nurse at a primary care unit

 Yes, most recently with a general practitioner on-call

 Yes, most recently with a private practitioner

 Yes, most recently with a school physician or nurse

 Yes, most recently with the paediatric emergency department

 Yes, most recently with another specialized physician: ____________

1. How was the most recent healthcare contact for your child made?

 Medical visit with a physician’s assessment

 Medical visit without a physician’s assessment

 Telephone contact

1. Were you advised to go to the paediatric emergency department at your most recent healthcare contact?

 Yes  No

1. Why are you visiting the paediatric emergency department today?

 I was advised by another healthcare provider to go here

 I did not know where else to go

 The paediatric emergency department is always open

 There is always a paediatric physician on call

 I have no good experience of primary care visits

 Other: __________________________________________

1. Would you still have come today if an appointment for your child had been offered

at the paediatric clinic tomorrow?

 Yes  No

in a primary care setting or at a private physician´s office tomorrow?

 Yes  No

at the paediatric clinic within a week?

 Yes  No

in a primary care setting or at a private physician´s office within a week?

 Yes  No

1. To what primary care health center does your child belong? ____________
2. In what country was your child born? ________________________________
3. In what country was your child’s father born? __________________________
4. In what country was your child’s mother born? _________________________
5. What languages are spoken in your child’s home? ______________________
6. How well does the child’s father understand, speak, read and write the Swedish language?

**Does not understand Swedish Understands all spoken Swedish**

     

**Cannot speak Swedish Speaks Swedish fluently**

     

**Cannot read Swedish texts Reads Swedish texts with no limitation**

     

**Cannot write in Swedish Writes in Swedish with no limitation**

     

1. How well does the child’s mother understand, speak, read and write the Swedish language?

**Does not understand Swedish Understands all spoken Swedish**

     

**Cannot speak Swedish Speaks Swedish fluently**

     

**Cannot read Swedish texts Reads Swedish texts with no limitation**

     

**Cannot write in Swedish Writes in Swedish with no limitation**

     

1. What is the father’s main activity?

 Working full-time

 Working part-time

 Not working

 Studying

1. What is the mother’s main activity?

 Working full-time

 Working part-time

 Not working

 Studying

1. What is the father’s highest educational degree?

 Elementary school (number of years ______ )

 Highschool

 University

 Other ______________________________________________

1. What is the mother’s highest educational degree?

 Elementary school (number of years ______ )

 Highschool

 University

 Other _________________________________________________

1. How many children are there in the family?

The family has _____ children, and this child is number _____ in siblings’ order.

1. How do the child’s parents live?

 Together  Separately

**Thank you for your participation!**
