# Supplementary material for: Influence of social characteristics on use of paediatric emergency care in Sweden - a questionnaire based study
Source: BMC Emerg Med. 2018 Dec 27;18:59. doi: 10.1186/s12873-018-0210-5 (PMC6307227; doi:10.1186/s12873-018-0210-5)
Supplement: Supplementary file 2 — Protocol on medical appropriateness of individual patient visits triaged for assessment - (to be filled out by paediatric emergency physicians). (DOC 35 kb) [file 12873_2018_210_MOESM2_ESM.doc]

**Protocol, physician**

**Please, answer the questions below after assessment of each child,**

**except for scheduled ED re-visits or ED arrivals between 21:00 and 07:59.**

**Social security code (patient): _________________________ Date: _____________**

**1.   What is your level of clinical profession (tick one alternative)?**

☐ Senior specialist of paediatrics (specialist experience ≥ 5 years)

☐ Specialist of peadiatrics (specialist experience < 5 years)

☐  Resident in paediatrics (paediatric experience ≥ 2 years)

☐ Resident in paediatrics (paediatric experience < 2 years)

☐ Resident in infectious medicine, general medicine or children psychiatry

☐  Intern physician (Swedish internship program)?

☐  Intern physician?

**2. Was the patient in need of a physician’s assessment?**

☐ Yes, by a paediatrician, or another physician supported by paediatrician,

at the ED

☐ Yes, by a general practitioner in primary care

☐ Yes, by another specialist ( ___________________ )

☐ No (end questionnaire here!)

**3.   The patient was in need of physician’s assessment (in relation to arrival time)**

☐ immediately

☐ not immediately but within 1 hour

☐ within 1-3 hours

☐ within 3-6 hours

☐ within 6-12 hours

☐ within 12-24 hours

☐ within 1-7 days

**THANK YOU FOR YOUR HELP!**
