# Supplementary material for: Influence of social characteristics on use of paediatric emergency care in Sweden - a questionnaire based study
Source: BMC Emerg Med. 2018 Dec 27;18:59. doi: 10.1186/s12873-018-0210-5 (PMC6307227; doi:10.1186/s12873-018-0210-5)
Supplement: Supplementary file 3 — Table S1. Patient visits and parental social characteristics at a large urban peadiatric emergency department (ED) in southern Sweden. Proportions and corresponding multiple regression analyses are reported in Table 2. (DOCX 24 kb) [file 12873_2018_210_MOESM3_ESM.docx]

**^Additional file 3: Table S1. Patient visits and parental social characteristics at a large urban peadiatric emergency department (ED) in southern Sweden. Proportions and corresponding multiple regression analyses are reported in Table 2.^**

| **Parental social characteristics^a^** | **Number (%) of** | | | | | | |  |
| --- | --- | --- | --- | --- | --- | --- | --- | --- |
|  | **included**  **patient^b^**  **visits at the paediatric ED** | **patients triaged away**  **from ED^c^** | *missing data^d^ from parental questionnaire* |  | **included patient^e^ visits triaged away from the ED or with physician protocol** | **patients considered appropriate for ED care** | *missing data^g^ from parental questionnaire* | |
| **Origin** | 923 (100) |  | *39 (4)* |  | 847 (100) |  | *35 (4)* | |
| One or more parents born in a Nordic country | 555 (60) | 67 (12) |  |  | 512 (60) | 261 (51) |  | |
| Both parents born in a non-Nordic country | 368 (40) | 80 (22) |  |  | 335 (40) | 122 (36) |  | |
| **Understanding of Swedish language** | 919 (100) |  | *43 (5)* |  | 848 (100) |  | *34 (4)* | |
| At last one parent with good understanding of  Swedish language | 837 (91) | 120 (14) |  |  | 770 (91) | 365 (47) |  | |
| Both parents having low^h^ understanding of  Swedish language | 82 (9) | 23 (28) |  |  | 78 (9) | 21 (27) |  | |
| **Level of education** | 924 (100) |  | *38 (4)* |  | 848 (100) |  | *34 (4)* | |
| At least one parent educated at least 12 years | 556 (60) | 84 (15) |  |  | 510 (60) | 246 (48) |  | |
| Both parents educated less than 12 years | 368 (40) | 59 (16) |  |  | 338 (40) | 140 (41) |  | |
| **Employment** | 930 (100) |  | *32 (3)* |  | 854 (100) |  | *28 (3)* | |
| At least one parent employed | 798 (86) | 117 (15) |  |  | 731 (86) | 343 (47) |  | |
| Both parents unemployed | 132 (14) | 28 (21) |  |  | 123 (14) | 43 (35) |  | |
| **Social status** | 920 (100) |  | *42 (4)* |  | 844 (100) |  | *38 (4)* | |
| Two-parent household | 788 (86) | 127 (16) |  |  | 726 (86) | 338 (47) |  | |
| Single-parent household | 132 (14) | 14 (11) |  |  | 118 (14) | 47(40) |  | |
| **Patient’s order of birth** | 890 (100) |  | *72 (8)* |  | 819 (100) |  | *63 (7)* | |
| First-born | 479 (54) | 89 (19) |  |  | 451 (55) | 196 (44) |  | |
| Not first-born | 411 (46) | 47 (11) |  |  | 368 (45) | 184 (50) |  | |

^a^ According to parental questionnaire.

^b^ Number of patients answering the specific questions in parental questionnaire and valid percent.

^c^ Number of patients triaged away from the ED and valid per cent of subgroup patients of parental social characteristics^a^.

^d^ Answers missing in parental questionnaire and percent of total number of study patients (n= 962).

^e^ Number of patients answering the specific questions in parental questionnaire and valid percent out of 882 children (80 children missing physician protocol).

^f^ Number of patients assessed appropriate for the paediatric ED according to structured assessments by ED physicians and valid per cent of subgroup patients of parental social characteristics^a^.

^g^ Answers missing in parental questionnaire and per cent of 882 children (80 children excluded missing physician protocol).

^h^ Self-rated as levels 1 - 4 on a six-level Likert scale.
